# Greenwash and green brand equity: The mediating role of green brand image, green satisfaction, and green trust, and the moderating role of green concern

**DOI:** 10.1371/journal.pone.0277421

**Published:** 2022-11-10

**Authors:** Minh-Tri Ha

**Affiliations:** 1 School of Business, International University, Ho Chi Minh City, Vietnam; 2 Vietnam National University, Ho Chi Minh City, Vietnam; Universita degli Studi di Perugia, ITALY

## Abstract

This study explores whether and, if so, how efficiently consumers’ greenwashing (GW) influences green brand equity (GBE) by integrating the mediation role of green brand image (GBI), green satisfaction (GSA), and green trust (GTR) and the moderating role of green concern (GC), using the legitimacy and signaling theories. A quantitative study was conducted by means of a questionnaire-based survey using a cluster random sampling technique with a sample of 436 consumers who purchased electronic products in supermarkets in Vietnam. A partial least squares structural equation modelling (PLS-SEM) approach was used to analyze data. Our findings show that GW is not significantly associated with GBE, possibly due to the halo effect. The findings also show that GW is adversely related to GBI, GSA, and GTR, which would positively influence GBE, except for the GW-GSA relationship. This means that, although GW does not directly affect GBE, it does indirectly influence it via GBI and GTR. Furthermore, GC significantly moderates the relationship between GW and GBE. Our work is the first to combine GBI, GSA, and GTR as mediators, and GC as a moderator using PLS-SEM approach to advance the theory of green brand equity on green marketing and to contribute significantly toward a unified theory of brand equity. Furthermore, our findings extend our understanding regarding the different mechanisms for which GBI and GTR play as mediators, and with GC as a moderator in the GW-GBE relationship in the electronics products setting of Vietnamese consumers.

## Introduction

In the new environmental age, firms are keen to find new approaches of applying green marketing to sell their products in the environmental era, so they are paying attention to environmental sustainability [1]. As the environmental challenges worsen, businesses and consumers are more interested in green consumption [2, 3]. Offers for greener products, for example, rose by 73 percent between 2009 and 2010 [4]. A growing demand for green products, combined with increased awareness of environmental challenges, is motivating businesses to act in an eco-friendly manner, whether through the implementation of eco-friendly management systems or the promotion of green marketing and branding [5]. In the projected period, the worldwide market of Green Technology and Sustainability is expected to expand from USD 11.2 billion in 2020 to USD 36.6 billion by 2025, representing a growth rate of 26.6 percent per annum [6]. Environmental awareness is expanding, as is interest from consumers and industry in green energy resources. A combination of these factors is propelling the market’s uptake of green technology, and sustainability-related products and services today [6]. Consequently, it appears that being green has become a success mantra for many businesses. Green marketing methods have been developed, with advertising slogans such as “environmentally friendly,” “green,” and “earth-friendly” being used to encourage customers to “go green” and “save the planet” in order to gain a competitive edge and attract eco-conscious customers.

Greenwashing is growing more prevalent, and most consumers have preconceived notions about firms and mistrust advertising, making it more challenging to believe in the credibility of eco-friendly marketing [7]. Consequently, trust may be harmed since consumers frequently base their purchase decisions on advertising and the business message [8]. Consumers may become more confused as a result of the erosion of trust since they do not know who or what to believe. Consumer mistrust, along with the perception of deceit, has had a detrimental influence on an organisation’s reputation and performance [9]. Furthermore, it will have a negative impact on customer buying intent.

With a fast rising economy and a population of more than 96 million [10], Vietnam is regarded one of the most promising markets. Vietnamese customers are becoming increasingly concerned about their health; thus they are focusing on safe products and healthy lives. As a result, analysts predict that “green products” will be the consumption trend of 2019 and the coming years [11, 12]. According to the Nielsen Vietnam report, up to 86% of Vietnamese consumers are prepared to pay more for green items, with a further 79 percent wanting to pay more [13]. The Vietnamese electronics industry has benefited from the country’s rapid economic growth and tech-savvy young population; retail sales of consumer electronics rose by 13.8 percent annually between 2014 and 2018, showing that the sector is prospering [14]. While Vietnam has an electronics market which has a high potential, it is also prone to deceptive advertising and greenwashing activities. Greenwash (GW) is characterized as “the act of misleading consumers regarding the environmental practices of a firm or the environmental benefits of a product or a service”; this is among the most widely referenced statements in the literature [15]. Consumers typically base their decisions on corporate advertisements and messaging, and greenwashing is eroding consumer trust in the advertising [16]. Consumers are unable to make green buying decisions because they do not know who or what to trust if they do not have a solid belief in the green claims of the firms. Consequently, greenwashing could jeopardize the entire green purchase market, as well as harming virtuous firms’ green marketing. Greenwashing continues to expand as the demand for eco-products constantly increases, despite the fact that customers are becoming more aware of this phenomenon [17]. As a result, consumers are also becoming highly skeptical with regard to businesses that benefit from environmental trends [18, 19].

Social identity and brand equity are essential for business success. Both academia and practice have deemed brand equity—the value which a logo or a brand name adds to a product [20]—to be of utmost importance [21]. Consumer perception of the brand is reflected in brand equity [20]. Consumers’ trust and confidence in the brand are influenced by brand equity, which in turn influences their intention to repurchase. Additionally, brand equity improves the effectiveness of the marketing initiatives [22]. Greater brand equity can enable businesses to increase sales, cut costs, increase profits, and implement an efficient marketing mix [20, 23]. Due to the increased focus on global warming, consumers are becoming more concerned with environmental issues [24]. They are more inclined to choose eco-friendly products [2, 25]. Hence, more businesses use “greenwashing” to trick consumers into thinking that they are environmentally friendly in an effort to boost their brand perceptions and boost customer satisfaction [26, 27]. Due to the increasing demand for green purchases, greenwashing is more common when businesses acquire green opportunities [28]. While previous studies have emphasized the related issues about brand equity [see, for example, 29, 30], none of these issues has been examined from the standpoints of greenwash, brand image, satisfaction, trust, and concern on environmental issues in a systematic manner. Consequently, this research would like to address this knowledge gap. Since businesses engage in more GW practices which damage consumers’ trust, this study suggests green trust (GTR) as the novel construct, and incorporates the concepts of green brand image (GBI), green satisfaction (GSA) and green brand equity (GBE), as proposed by [1], into a more comprehensive framework to address the effect of GW on GBE in the green marketing domain. Additionally, businesses’ greenwashing practices may result in a decrease in GBE, which is influenced by consumer attributes, one of which is green concern [31]. Environmental or green concern (GC) is often regarded as an antecedent to green brand equity [2, 32]. In this study, we propose that GW has a detrimental influence on GBE and GBI, GSA, and GTR mediate the GW-GB relationship, while GC acts as a moderator.

In the face of strict foreign environmental legislation and widespread consumer environmentalism, GBE is critical for businesses. Our study explores whether and how consumers’ views of GW affect GBE by integrating the mediation role of GBI, GSA, and GTR and the moderating role of GC, based on the legitimacy and signaling theories. We propose a model to study the mediation role of GBI, GSA, and GTR and the moderating role of GC. Our proposed model is in line with environmental trends to assist businesses in improving their GBE and to advance the green marketing literature.

Prior research studying greenwash and green brand equity is very limited and uses different methodologies. For example, [1] uses regression method, and [33] uses covariance-based structural equation modeling (CB-SEM). This study attempts to use partial least squares structural equation modeling (PLS-SEM) to study greenwash and green brand equity for several reasons: given the need for a unifying brand equity theory, PLS-SEM is appropriate for exploratory research for theory development [34] in which, for the purpose of this study, green brand image, green satisfaction, and green trust play a mediating role and green concern plays a moderating role between greenwash and green brand equity. This gives important evidence toward a unified theory of brand equity [35]. Until now, no research has applied this method in the proposed context. Regression has previously been used to study greenwash and green brand equity [1], while the study that investigated greenwash and green brand equity with information and knowledge as moderator used the CB-SEM method [33]. Therefore, our study is unique in both its nature and its methodological approach.

While previous studies have highlighted the related issues about (green) brand equity [2, 25, 32, 36, 37], none of these issues has been examined from the standpoints of greenwash, brand image, satisfaction, trust, and concern in environmental issues systematically. This study also responds to a call from [1] for research to be carried out “on other countries in comparison with this study.” Consequently, this research would like to bridge this knowledge gap. To achieve this, we develop a research model of green brand equity based on signaling theory and legitimacy theory that are both in line with environmental trends to assist businesses in improving their green brand equity and to advance the green marketing literature.

Our research makes several contributions. First, in contrast to conventional research, which concentrates on investors and stakeholders in general, our work, which is grounded in legitimacy and signaling theories, responds to the recent call for research into the consumer repercussion of greenwashing [38, 39] by investigating GW and its role in GBE. Second, our study extends and advances our understanding of the mechanism in which GW influences GBE. More precisely, our work explores the mediating effects of GBI, GSA, and GTR in the GW-GBE relationship, as well as the moderating effect of GC. Although the extant literature has seen GC acting through an antecedent [2, 32], it has not done so in a mediating or moderating role; our work validates this role. Furthermore, we integrate GBI, GSA, and GTR as mediators into the research framework to study the relationship between GW and GBE in a more systematic manner. Finally, contrary to studies conducted in developed countries [see, for example, 5, 40], our work investigates GW in a developing country, namely, Vietnam in this study, whose environment is declining as a result of a lack of sound regulations despite a strong economic growth, and contributes toward a unified theory of brand equity [35].

## Literature review and hypotheses development

### Background theories: Corporate legitimacy and signaling theories

Our research examines the effect of GW, GBI, GSA, and GTR on GBE using corporate legitimacy and signaling theories. The greenwash phenomenon was associated with the literature of corporate legitimacy [41]. The literature differentiates the following three main kinds of corporate legitimacy: cognitive, moral, and pragmatic legitimacy [42–44]. Cognitive legitimacy may “involve either affirmative backing for an organisation or mere acceptance of the organisation as necessary or inevitable based on some taken-for-granted cultural account” [44]. Moral legitimacy considers “a positive normative evaluation of the organisation and its activities,” and is thus based “on judgments about whether the activity is the right thing to do” [44]. On the contrary, pragmatic legitimacy “rests on the self-interested calculations of an organisation’s most immediate audiences” [44], and it is thus derived from the perceptions of stakeholders regarding their personal benefit which stems from organizational communication and activities. In a similar vein, GW happens in the setting of pragmatic legitimacy with respect to the legitimacy theory [43, 45, 46]. Greenwashing is based on the perceptions of stakeholders as to how genuine the corporate green communication actually is. Inarguably, pragmatic legitimacy may be associated with the practice of deliberately deceiving stakeholders’ perceptions about environmental and social activities. As a matter of fact, the strategic approach of adopting a managerial viewpoint legitimately, where legitimacy is dependent on the organizational capability to exploit symbols instrumentally in order to gain social acceptance, can be used to handle pragmatic legitimacy. Specifically, the strategic approach focuses on how organisations can use symbolic communication to strategically manipulate the stakeholders’ perceptions [47].

On the other hand, signaling theory concentrates on the communication of information in order to disclose favorable firm features [48], and it suggests that brands are an effective means for customers and firms to determine quality [49]. Signaling theory aims to decrease the information asymmetry between both parties [50], and this emphasizes the importance of credibility in determining consumer-based brand equity [49]. Taking the marketing into consideration, these parties are typically a buyer and a seller. Normally, a signaler (for example, a product, a person, or a firm) provides a signal to a receiver (for example, an individual or a firm) who, in turn, delivers feedback to the signaler [51]. Signaling is most helpful for products where the quality is either uncertain or undisclosed until purchase [52]. Using signaling theory and key assumptions of asymmetric information [50, 51, 53, 54], [43] claim that there are two key reasons why untrue (or deceptive) green communication can be viewed as an effective signaling approach for misleading or distorting the view of stakeholders. First, it is beneficial that low-performing organisations reap both environmentally and socially from signaling their contribution or commitment to eco-friendly matters. In addition, as far as signaling theory is concerned, [51] argue that every organisation possesses the potential as to whether or not to signal its authentic quality externally. This implies that organisations with a low performance might see signaling their (falsified) message as a source of high motivation (due to the anticipated legitimacy gains) with respect to costs. As a result, stakeholders are unable to differentiate between tangible and strictly symbolic values based on a firm’s involvement in an environmentally friendly communication [55] or green strategies [56]. Second, communication depends on an information asymmetry between the receiver and the signaler, which is why corporations are so effective at sending false signals. The aim of signaling theory is to diminish information asymmetry between the information holder and the information receiver [50]. [57] noted that the “scientific knowledge required to understand issues underlying many environmental claims is often complex and subject to change, making it difficult for the general public to decipher what is actually being said.” As a consequence of the information asymmetry between a firm and its related stakeholders, false green messages can be used as a signal of a corporate eco-friendly action or behavior.

Building on corporate legitimacy and signaling theories, our work analyzes the mediating roles of GBI, GSA, and GTR, and the moderating role of GC in the GW-GBE relationship.

### GW and GBI

In the era of sustainable development, when consumers’ demands for green businesses are increasing, and regulations are becoming increasingly tight, it is only beneficial for business firms to be looked at as green brands or to have positive GBIs [2, 58]. [59] characterizes GBI as “a set of perceptions of a brand in a consumer’s mind that is linked to both environmental commitments and environmental concerns.” GBI is important for firms since it increases green awareness [60], has a favorable influence on green brand preference [61] and is positively associated with green brand loyalty [62]. More importantly, GBI is found to link directly to green purchase behavior [58, 63] and contributes to green competitive advantages [64].

GBI also covers three types of benefits: functional, symbolic, and experimental, which [62] call “green benefits”. [64], on the other hand, classify these benefits into whether they are tangible or psychological. These authors assume that both benefits are equally important. According to [62] and [65], the greatest effects for brand positioning come from reasonable combinations of the two types. Greenwashing uses exaggerated or false advertising about the greenness of products or brands, making consumers skeptical about green claims, and greenwashing may negatively impact GBI [62].

Consumers may now analyze and assess a firm’s environmental information disclosure more easily in the social network and information technology sectors, overcoming the disadvantage of asymmetric communication between firms and consumers, and detecting wrongdoers [38, 66]. Firms utilizing greenwashing to promote a positive brand image may use a different tactic because this one is not effective [66]. [63] argue that greenwashing has made consumers skeptical about green claims, confusing them with green marketing, and having increasingly negative attitudes toward a firm’s environmental campaigns, thus damaging a firm’s GBI. Greenwashing is an obstacle to expanding green marketing approaches [67]. Several empirical studies found that greenwashing impacts negatively on GBI [1, 63, 68, 69]. Hence, it is proposed that greenwash has a negative impact on green brand equity.

*Hypothesis 1 (H*_*1*_*)*: *GW has a negative impact on GBI*.

### GW and GBE

GBE is a recently-developed terminology that integrates the characterization of brand equity and environmental associations. [59] characterizes GBE as “a set of brand assets and liabilities about green commitments and environmental concerns linked to a brand, its name and symbol that adds to or subtracts from the value provided by a good or a service.” As with brand equity, GBE refers to the added value that brands have on physical items [2, 70]. Consumers are perceived to prefer a product with a higher brand equity over another product with the same functions. In relation to green products, “added value” relates to the attributes of products that satisfy consumers’ needs for environmental protection. [71] contend that products with higher GBEs will be highly prioritized by consumers.

According to [72], false sustainable communication results in high risks for firms. Taking examples from the cases of Shell, BP (British Petroleum), ExxonMobil and Nestlé, the study by [72] shows that exaggerated sustainable claims could backfire on the firms if there was a discrepancy between the “talk” and the “concrete action”, which might subsequently damage their image and reputation [73]. Greenwashing is also a relative concept for which [74] have developed a greenwash index to examine how it impacts on GBE. They further find that, the more that the firms are perceived to be greenwashing, the more their brand equity is reduced. Additionally, [1, 40, 75] find that greenwashing has negatively impacted either directly or indirectly on GBE through mediators such as brand association, brand credibility, GTR, green confusion, and green perceived risk. Hence, greenwashing is not economically efficient. If firms do not want to have their reputational assets destroyed, green claims must be verified by actual behaviors. Therefore, it is proposed that greenwashing has a negative impact on GBE.

*Hypothesis 2 (H*_*2*_*)*: *GW has a negative impact on GBE*.

### GW and GSA

According to [59], GSA is “a pleasurable level of consumption-related fulfilment to satisfy a customer’s environmental desires, sustainable expectations, and green needs.” Consumer satisfaction is determined by comparing expectations with experiences of product performance. Consumers are satisfied if the product offers features that meet their expectations. By contrast, dissatisfaction occurs if products are perceived to disconfirm consumers’ expectations negatively. Moreover, GSA relates to consumers’ overall confirmation and positive disconfirmation in the way that a product promotes how it is environmentally friendly [2, 76].

Consumer satisfaction is an important construct which represents the closeness of the relationship between firms and consumers. Classically, higher satisfaction is a positive indicator for the long-lasting relationships which consumers have with firms. It predicts consumers’ purchase behaviors. Consumers who are happier with the items, for example, are more inclined to repurchase them. Satisfaction also brings positive word-of-mouth (WOM) that helps firms to attract new consumers [77]. Hence, investing in satisfying consumers is a wise decision for all firms.

Many green claims now overstate a product’s green functionality or attributes, so this may reduce consumer satisfaction. [77] confirmed the adverse impact of GW on GSA regarding Taiwanese consumers who purchased electronics products. Likewise, greenwashing significantly reduces the satisfaction-loyalty relationship [1, 77, 78]. In a similar vein, Martínez, Cremasco [79] and [1] confirm the adverse association between GW and GSA. Therefore, it is hypothesized that greenwash has negative impact on GSA.

*Hypothesis 3 (H*_*3*_*)*: *GW has a negative impact on GSA*.

### GW and GTR

GTR is important for green marketing [80], because together with credibility, trust plays an essential role for green marketing communications [2, 59, 70]. Consumer trust gives consumers sufficient confidence in green advertising to make purchasing decisions [81]. GTR is found to be positively related to GBE [2, 25, 59].

Product harm crises such as a smartphone catching fire may impact individuals and are less likely to cause severe damage to brand trust than greenwashing. Greenwashing is considered to be an environmental crisis, but it is also sometimes a social problem because it causes a negative impact on the public. If firms are accused of greenwashing, their reputations and brand trust will also be hurt [82, 83].

Nowadays, the market is flooded with green information and green claims. Consequently, this confuses consumers and they rarely believe these claims. The study by [81] finds that GW is used to increase perceived risk as well as the confusion of a consumer, and is negatively related to GTR. According to [83], trust between two parties increases when consumers’ knowledge about the green claims’ intent or quality increases. However, greenwashing reduces consumer attitudes about firms’ environmental commitments, and thus consumers eventually no longer believe in green marketing. Based on the Trust-Based Marketing Theory, it is advised that the hotel industry should reduce greenwashing practices to enhance consumers’ trust [83]. Hence, we propose that greenwashing has a negative impact on GTR.

*Hypothesis 4 (H*_*4*_*)*: *GW has a negative impact on GTR*.

### GW, GBE, GBI, GSA, and GTR

Brand equity is a collection of assets that includes four components: brand salience, brand meaning, brand responses, and brand resonance [20]. Brand equity generates added value to a product, making it more favorable to the other products in the consumer’s perspective. On the one hand, the higher the brand equity, the higher the prices because consumers will be willing to pay more [59]. On the other hand, the businesses which own strong brands will have great advantages. Specifically, a strong brand provides the “brand platform” for a firm to extend its product line, or license the brand or “brand resiliency”, to reduce the adverse impact on the firms and to recover quickly from crisis situations, and also “brand dominance”, which enables a firm to differentiate itself from its competitors and gain dominance in the market [20, 31]. Furthermore, a strong brand relates to a firm’s increasing sales, efficient operation, and cost savings [84], and also provides consumers with economic and emotional benefits because it saves them time and reduces the perceived risk [85].

In the highly competitive green market, green brands are more important for business firms. Green brands improve consumer attitudes toward the brands and influence their behavior. For example, a green brand positioning has a favorable effect on attitudes about green brands which, in turn, has a favorable influence on green purchase intention [86, 87]. Green brands also result in positive green WOM communication [71]. Positioning as a green brand helps firms to differentiate their products and achieve competitive advantages [36, 88]. Therefore, GBE is also an asset of a firm which can affect the consumers’ perceptions of green products, making one green product effectively more worthy than the rest [2, 25, 70, 71]. Because of this increase in public environmental concerns, GBE is gaining more attention from firms [2, 70, 89].

Since the introduction of GBE in 2010, several scholars and researchers have focused on investigating the factors affecting GBE which include GBI, GSA, and GTR [2, 25, 33, 59]. This includes the mediating role of GBI, GSA, and GTR in the relationship between GW and GBE [33]. Several studies find that GBI is positively related to GBE [1, 2, 25, 33, 37, 59, 71]. Similarly, GSA is found to be positively associated with GBE [1, 33, 59]. Finally, GTR is confirmed to be positively associated with GBE [2, 25, 32, 33, 71]. Consequently, the following hypotheses are formulated below:

*Hypothesis 5 (H*_*5*_*)*: *GBI has a positive impact on GBE*.
*Hypothesis 6 (H*_*6*_*)*: *GSA has a positive impact on GBE*.
*Hypothesis 7 (H*_*7*_*)*: *GTR has a positive impact on GBE*.
*Hypothesis 8 (H*_*8*_*)*: *GBI has a positive impact on GSA*.
*Hypothesis 9 (H*_*9*_*)*: *GTR has a positive impact on GSA*.
*Hypothesis 10 (H*_*10*_*)*: *The influence of GW on GBE is mediated by GBI*.
*Hypothesis 11 (H*_*11*_*)*: *The influence of GW on GBE is mediated by GSA*.
*Hypothesis 12 (H*_*12*_*)*: *The influence of GW on GBE is mediated by GTR*.

### The moderating role of GC

The term “green” or “environmental concern” refers to an individual’s stated active intention to safeguard the environment [90]. Although several studies regard this concern as an antecedent of GBE [2, 32], no research ever examines its moderating role between GW and GBE. Businesses understand that addressing consumers’ environmental issues increases consumer preference for their products or services [91]. In fact, environmentally conscious consumers are more inclined to recycle and buy green items [92–95]. Additionally, they are more likely to recognize corporate greenwashing and be aware of its adverse consequences, resulting in a decrease in the use of such items [96]. Additionally, we have seen GC as a moderator in several studies regarding consumers’ green purchasing intentions [see, for example, 97–99]. In Vietnam, people are paying increasing attention to protecting the environment [100]. With this concept in mind, and in light of the country’s severe pollution, Vietnamese consumers who are highly concerned about the environment are likely to impose their green views on their buying habits, therefore reducing their unethical purchasing intents [39]. We thus argue that GC moderates the relationship between GW and GBE. Therefore, we formulate a hypothesis as given below.

*Hypothesis 13 (H*_*13*_*)*: The association between GW and GBE is moderated by GC. The higher the GC is, the more adverse effect on GBE the GW will be.

**Fig 1:**
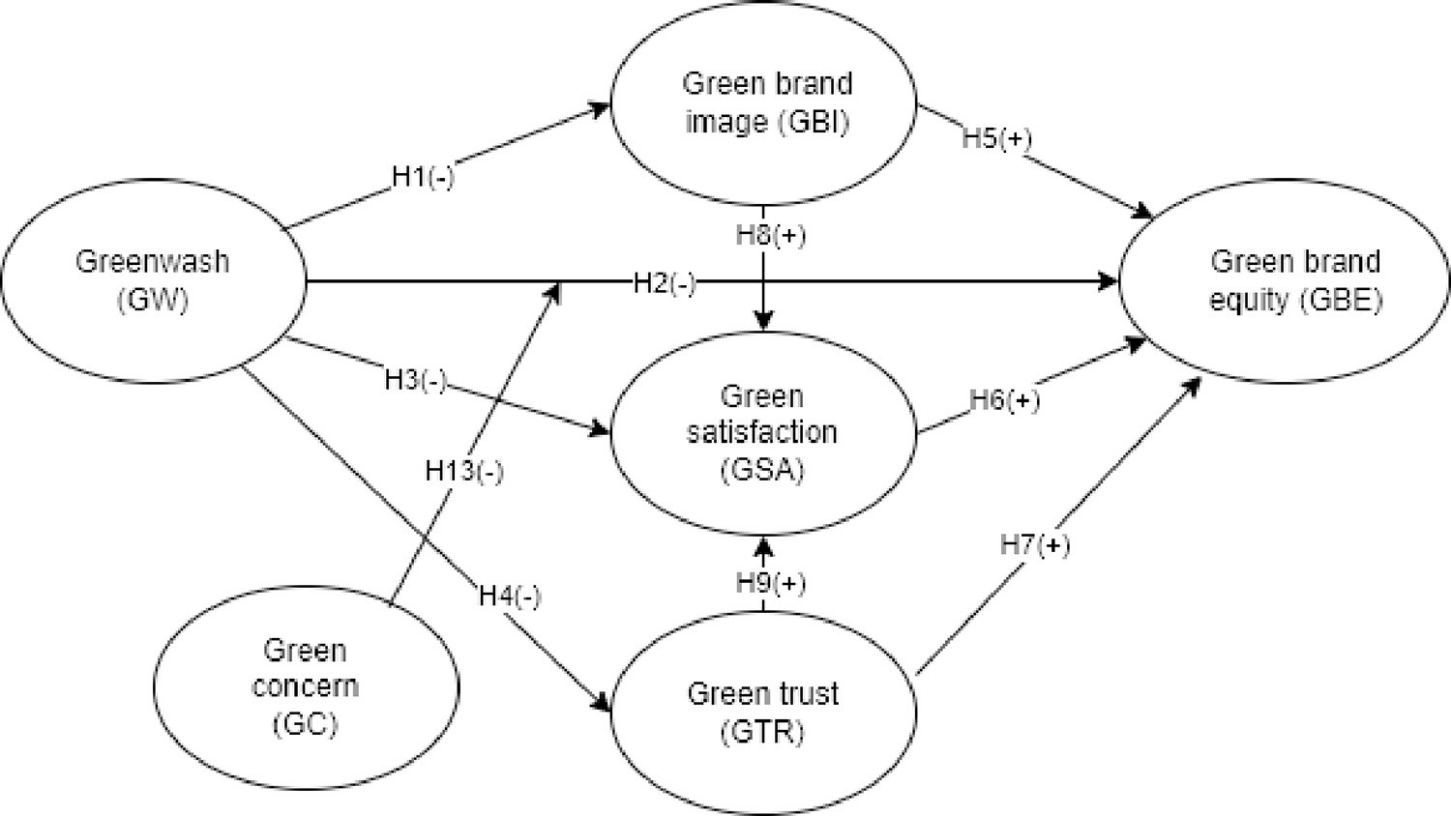
Hypothesized model.

This research hypothesizes that the GW adversely affects GBE, while GBI, GSA, and GTR mediate, and GC moderates the negative association between GW and GBE. Fig 1 exhibits the hypothesized model.

## Methodology

### Data collection and sample

This study’s research approach entails a cross-sessional survey carried out with electronic consumers in Ho Chi Minh City, the biggest and busiest city of Vietnam. The electronic consumers are those who have experience in purchasing the major home appliances which have the highest greenhouse gas emissions among household appliances [101], so the manufacturing and the design of these goods therefore have to follow strict international laws and regulations to minimize their environmental impacts [59]. Green consumers actively seeking environmentally friendly products [102] will tend to purchase those products to satisfy their environmental needs. Additionally, our research investigates the brands of electronic products that are big international products and are familiar to the Vietnamese. With a clear understanding about brands, it is comforting for consumers to participate in this research.

Face-to-face interviews with a small, representative sample of respondents are used to pretest the questionnaire (n = 10) prior to the data collection [103]. The purpose of this is to evaluate if any statements are too hard to comprehend because of unusual language, grammar or wording [104], thus establishing the construct validity of the questionnaire [105]. Results from the pretest show that respondents are confused about the term *greenwashing*, and they suggest that an explanation should be included in the questionnaire. We subsequently adopt an explanation of *greenwashing* and several examples of greenwashing in our questionnaire.

Our study collected a total of 550 responses between June and July of 2020 using the convenience and snowball sampling methods. Data was collected using both direct interviews and online questionnaires [103, 106]. The direct interviews and online questionnaires account for 63.6% and 36.4% of the responses, respectively. With clear instructions, the response rate is 79.8%, resulting in 436 valid responses. This response rate is high and helps to reduce response bias [107]. All of the respondents are between the ages of 18 and 60 with an average age of 32.1 years. Males account for 42.3% of the respondents, and females account for 57.7%. The sample size needed for the PLS-SEM must be “at least ten times the largest number of structural paths directed at a particular latent construct in the model” [108]. As seen in Fig 1, there are five paths (from GW, GBI, GSA, GTR, and GC) that lead to GBE, indicating that the sample size must be larger than 50. As a result, the sample of 436 responses in our investigation meets the PLS-SEM minimum size criterion.

### Measurement of constructs

Our research measures the items using five-point Likert scales that range from “1” (strongly disagree) to “5” (strongly agree). To establish content validity, measurements have been taken from the previous literature and adapted accordingly [109]. The scales for greenwashing, GBI, and GTR are measured with five items in each construct, and have been adapted from [33, 59, 81]. The scales of GSA and GBE are measured with four items in each construct, and they are all adapted from [33, 59]. The scale of GC is measured with four items and is adapted from [98].

#### Data analysis

We employ the PLS-SEM method to analyze our data. PLS-SEM has been widely used for many years [108] and requires neither a large sample size nor data normality [34]. The analysis consists of two steps as described by [34]. Step one is to evaluate an outer model in which reliability and validity must be satisfactory. Step two is to evaluate an inner model to generate loadings, and structural model relationships for the latent constructs and the observed variables. Finally, a bootstrap was carried out to evaluate the statistical significance of the hypothesized relationships in the model. In this particular study, we used SmartPLS version 3.3.3 for data analysis [110].

## Empirical analysis and results

### Evaluation of outer model

Before evaluating the outer model, we test common method bias (CMB) to see if it is a problem as all variables are measured using the same instrument. Harman’s single factor test is used as this is one of the most popular approaches for testing CMB [111]. The result indicates that the single factor accounts for 41.700% of the variance which is smaller than 50%. Consequently, CMB does not seem to be an issue in our analysis [112].

Table 1 summarizes the results of the outer model, including the outer loading, α, rho_A, CR, and AVE. During a reliability test, indicators GW5, GC3, GC4, and GSA1 were eliminated as they did not pass the cut-off point of 0.40 [113] as our study is exploratory in nature. Table 1 shows that the CR values vary from 0.775 (GW) to 0.932 (GBI), surpassing the requirement value of 0.70 [34]. Cronbach Alpha and rho_A surpass the cut-off values of 0.60. This means that all constructs satisfy internal consistency reliability.

**Table 1:** Constructs and their responding measures.

Construct	Code	Item	Loading	α	rho_A	AVE	CR
GBI	GBI1	1) The brand is commonly regarded as the gold standard in terms of environmental commitments.	.912	.908	.909	.732	.932
	GBI2	2) When it comes to environmental credibility, the brand is serious.	.882				
	GBI3	3) In terms of environmental sustainability, the brand is a success.	.847				
	GBI4	4) When it comes to environmental concerns, the brand is well-known.	.824				
	GBI5	5) The brand can be trusted when it comes to environmental commitments.	.809				
GTR	GTR1	1) The environmental reputation of this product is inherently trustworthy.	.851	.888	.890	.693	.918
	GTR2	2) The environmental performance of this product is reasonably reliable.	.864				
	GTR3	3) The environmental statements made by this product are generally reliable.	.743				
	GTR4	4) The environmental concern of this product meets my needs.	.850				
	GTR5	5) This product adheres to its environmental promises and commitments.	.847				
GSA	GSA1	1) This brand is my choice due to its environmental commitments. (d)		.750	.759	.667	.857
	GSA2	2) Because of its environmental credentials, purchasing this brand is a smart idea.	.813				
	GSA3	3) I am pleased with my purchase of this brand due to its eco-friendliness.	.775				
	GSA4	4) I like this brand because of its environmental consciousness.	.859				
GW	GW1	1) The brand uses terms to mislead its environmental characteristics.	.807	.685	.692	.615	.827
	GW2	2) The photographs or visuals used in the brand’s environmental elements are deceiving.	.822				
	GW3	3) A green claim made by this brand is unclear or seems to be unprovable. (d)					
	GW4	4) The brand overstates the extent to which it is green.	.719				
	GW5	5) The brand omits key details making the green claim seem more impressive than it really is. (d)					
GBE	GBE1	1) Even if the products are identical, purchasing this brand over others is worthwhile due to its eco-friendly commitments. (d)		.868	.870	.792	.919
	GBE2	2) I would prefer this brand even though another offered the same environmental benefits.	.889				
	GBE3	3) I’d buy another brand if its environmental quality was as strong as this brand’s.	.878				
	GBE4	4) If another brand’s environmental concerns aren’t significantly different from this brand’s, it seems better to buy this brand.	.902				
GC	GC1	1) I am concerned about the deterioration of the environment’s quality.	.925	.837	.837	.757	.903
	GC2	2) For me, the environment is a big source of worry.	.785				
	GC3	3) Environmental concerns are something that I am really enthusiastic about.	.894				
	GC4	4) I am always considering ways to enhance the overall state of the environment. (d)					

Note: (d) indicates that the measures fail the validity and reliability tests.

In terms of convergent validity, all AVEs range from 0.615 (GW) to 0.792 (GBE), indicating that the convergent validity is achieved. Additionally, the heterotrait–monotrait ratio of correlations (HTMT) is a new criterion for assessment of the discriminant validity [34]. The HTMT values should be below 0.85 [34]. Consequently, this study reveals that all HTMT values are less than 0.85, indicating that discriminant validity is achieved. The results can be found in Table 2. As a result, it is possible to conclude that the hypothesized model’s constructs are not only reliable and valid, but are also distinct from one another.

**Table 2:** Results of discriminant validity using HTMT.

	GBE	GBI	GC	GC*GW	GSA	GTR	GW
**GBE**							
**GBI**	0.831						
**GC**	0.847	0.733					
GC*GW	0.063	0.045	0.042				
**GSA**	0.539	0.493	0.422	0.047			
**GTR**	0.759	0.657	0.696	0.022	0.616		
**GW**	0.399	0.408	0.340	0.231	0.349	0.607	

Source: Author’s calculation.

### Evaluation of inner model

**Fig 2:**
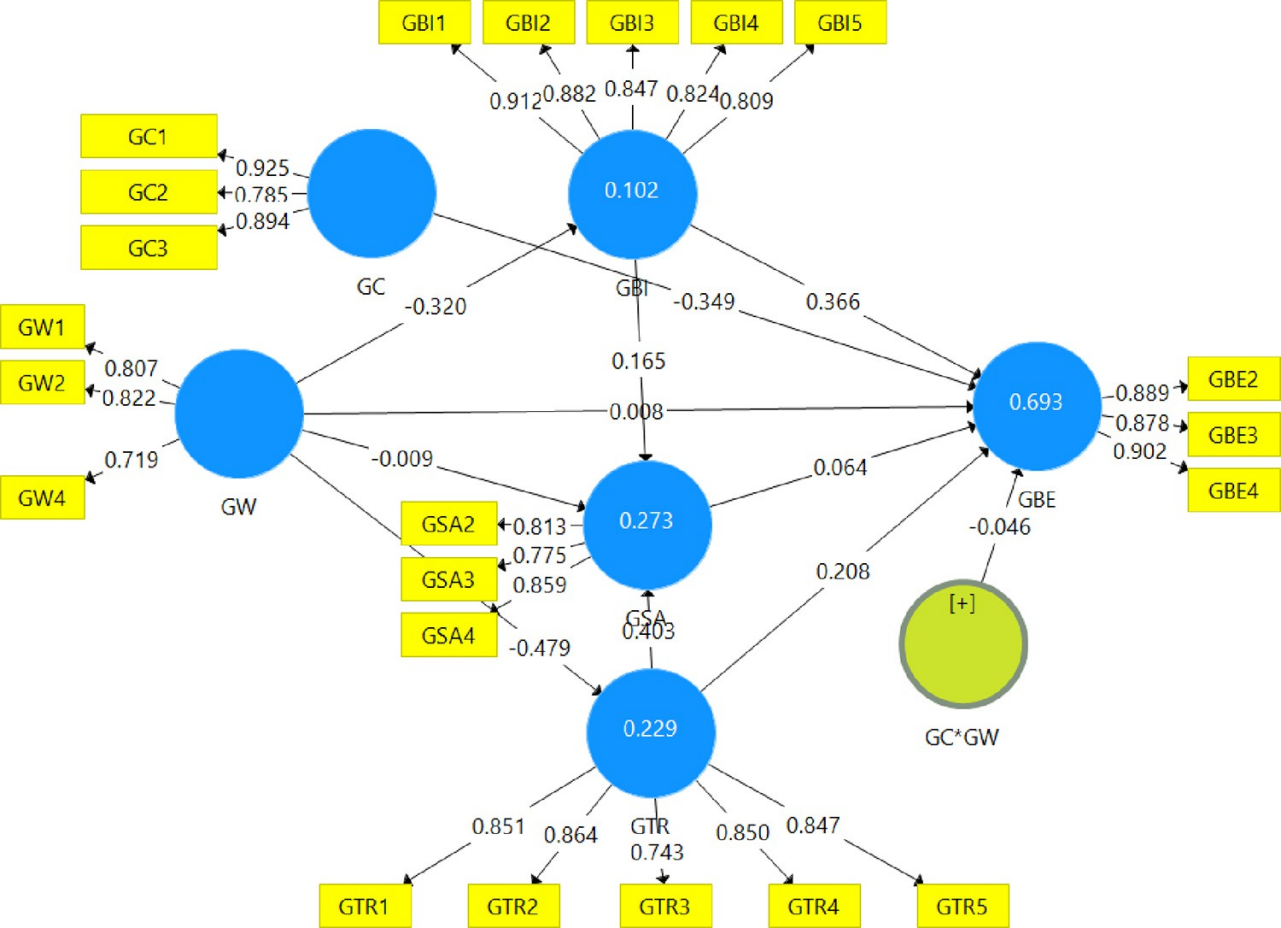
SmartPLS results.

The next step is to evaluate the inner model to verify the hypothesized relationships after assessing the outer model. Fig 2 presents the path coefficients for all variables and R-squares.

As illustrated in Fig 2, the path coefficients show the strengths of the constructs’ relationships, while the R-squared values show how much variation each construct has in the model. The bigger the R2 value, the more explanatory power the PLS structural model has, and thus the better the predictability of the endogenous constructs [114]. According to Hair, Risher (34), when the R-squared value reaches .75, .50, and .25, this means that the value is substantial, moderate, and weak, respectively. However, R-square may also vary depending on the research area. As shown in Fig 2, R-square (GBI) = .102, R-square (GSA) = .273, R-square (GTR) = .229, and R-square (GBE) = .693; therefore, these values imply that the research model’s explanatory power ranges from weak to relatively substantial.

In addition, our research also uses a 5000-sample bootstrap to verify the statistical significance of the hypothesized connections [115]. As illustrated in Table 3, except for H_2_ and H_3_, all relationships are validated statistically as all of the p-values are < .10.

**Table 3:** Bootstrapping results.

	Path Coefficient	Observed T-Statistics	Standard Deviation	Effect size (f2)	Bias	Confidence Intervals (2.5%)	Confidence Intervals (97.5%)	P-value	Results
**H1. GW → GBI**	-0.320	7.453	0.043	0.114	-0.003	-0.398	-0.232	0.000	Supported
**H2. GW → GBE**	0.008	0.247	0.033	0.000	0.001	-0.054	0.073	0.805	Not supported
**H3. GW → GSA**	-0.009	0.180	0.049	0.000	-0.002	-0.101	0.088	0.857	Not supported
**H4. GW → GTR**	-0.479	12.638	0.038	0.298	-0.002	-0.549	-0.399	0.000	Supported
**H5. GBI → GBE**	0.366	6.596	0.056	0.219	0.002	0.259	0.472	0.000	Supported
**H6. GSA → GBE**	0.064	1.670	0.038	0.010	0.000	-0.012	0.137	0.095	Supported
**H7. GTR → GBE**	0.208	4.848	0.043	0.061	-0.002	0.127	0.294	0.000	Supported
**H8. GBI → GSA**	0.165	3.007	0.055	0.025	0.000	0.058	0.274	0.003	Supported
**H9. GTR → GSA**	0.403	6.063	0.067	0.125	0.000	0.270	0.529	0.000	Supported
H13. GC*GW → GBE	-0.046	1.650	0.028	0.008	0.001	-0.100	0.010	0.099	Supported

Source: Author’s calculation.

### Moderating effects

**Fig 3:**
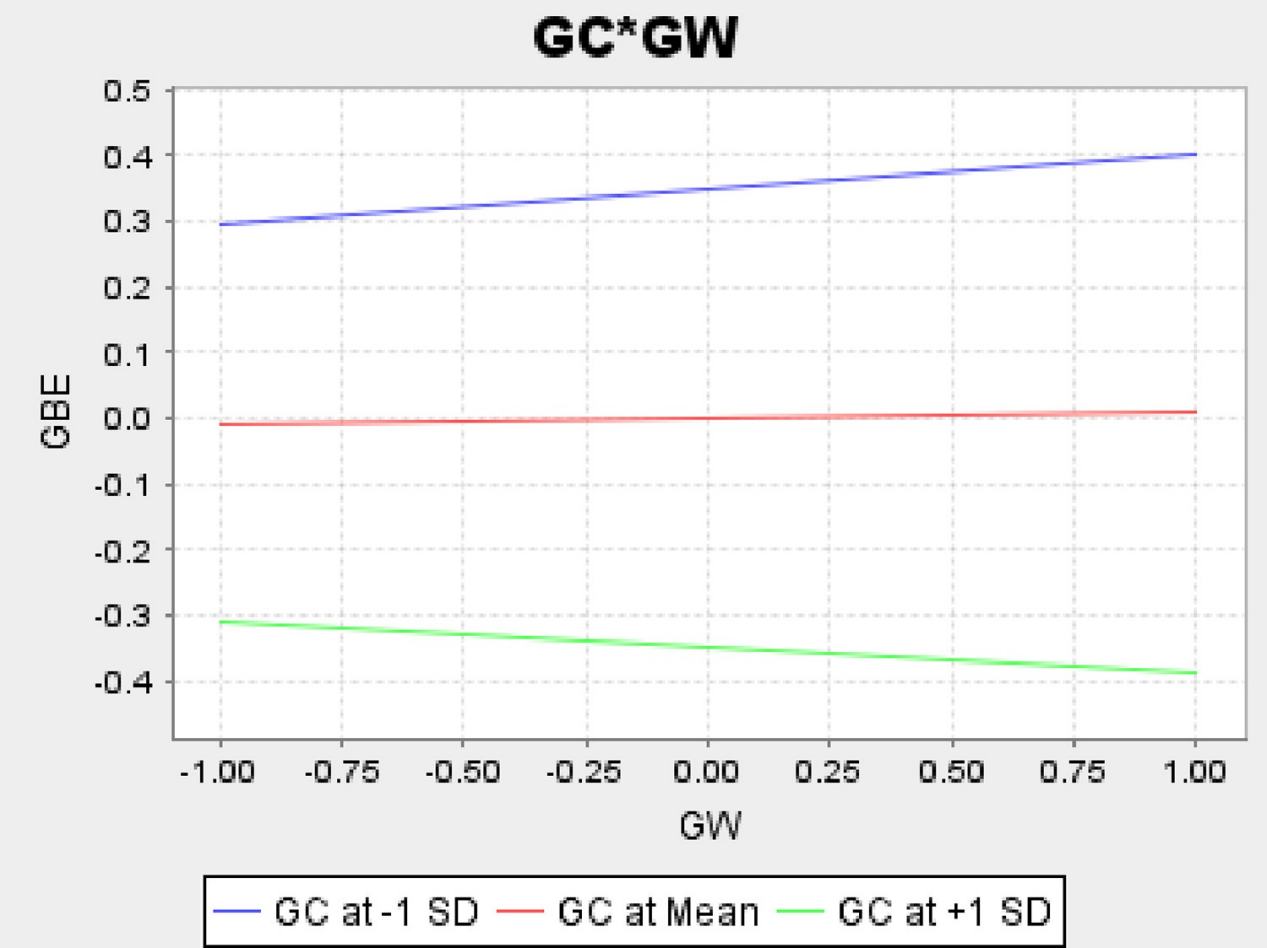
Moderating effect of GC on the GW-GBE relationship.

We hypothesized that the GW-GBE relationship is moderated by the presence of GC (H_13_). We found a significant negative effect of GC (β = -.349, ρ = .000) on GBE. The significant interaction effect (β = -.046, ρ = .099) of GC and GW on GBE indicates that the effect of GW on GBE is contingent upon GC. The slope of the line, displaying the association between GW and GBE, is greater for a lower GC as compared with a higher GC (Fig 3). Therefore, for a higher GC, the relationship between GW and GBE is weaker and vice versa. This provides empirical evidence for the moderating effect of GC.

### Mediating effects

Furthermore, we tested for the indirect effect to determine the role of GBI, GSA, and GTR constructs as mediators in the relationship of GW and GBE. As a result, indirect effects were calculated and shown in Table 4.

**Table 4:** Indirect effects analysis.

	Specific Indirect Effects	Observed T-Statistics	Standard Deviation	Bias	Confidence Intervals (2.5%)	Confidence Intervals (97.5%)	P-value	Results
**H10. GW → GBI → GBE**	-0.117	4.447	0.026	-0.002	-0.173	-0.072	0.000	Supported
**H11. GW → GSA → GBE**	-0.001	0.156	0.004	0.000	-0.009	0.006	0.876	Not supported
**H12. GW → GTR → GBE**	-0.100	4.559	0.022	0.000	-0.148	-0.060	0.000	Supported

Source: Author’s calculation.

In terms of indirect effects, Table 4 validates the role of GBI and GTR as mediators in the GW-GBE relationship.

## Discussion

Greenwashing is still common in industry, despite the fact that society deters it. As a result, greenwashing will jeopardize the growth of green marketing. Since many green claims on the market are misleading, greenwashing seems to be a significant barrier to green marketing [4]. Theoretically, little attention has been paid to consider systematically how GW can shape a connection between GW and GBE. Given the rising prevalence of GW practices, it is essential to develop a model that shows how greenwashing directly affects GBE and enables GBI, GSA, and GTR, which then influences GBE.

One finding that contradicts our expectation is that GW is not significantly associated with GBE (p = .697). This is an interesting and surprising finding, as it is inconsistent with previous studies [1, 40, 46]. This may be explained by the so-called “halo effect”. [116] characterizes the “halo error” (halo effect) as a “constant error toward suffusing the ratings of special features with a halo belonging to the individual as a whole”. The “halo effect” states that one element of an entity’s attributes overwhelm its other aspects [116]. Similarly, the “halo effect” has an impact on one entity due to its connection with another [117]. For example, the adverse feature of an entity may overshadow its positive feature, and vice versa [118]. This “halo effect” rule may be applicable to the market of electronics products in Vietnam [119]. Furthermore, [120] described that social status is a source of a “halo effect” as it enhances general consumers’ environmental feelings about firms, making the social evaluations become imprecise predictions of firms’ environmental performances. The “halo effect” has been used in consumer research to account for perceptual biases that consumers may have as a result of, for example, a prominent cue or signal, such as a product label [121, 122]. In our study, it may be the case that the electronic brands chosen can be considered to be the symbols of innovation and modernization about which the consumers are proud, and they are loyal and supportive to the brands of their choice. In addition, brands of electronic products in Vietnam seem to have large environmental campaigns which satisfy them and overcome mistakes in environmental advertising or trivial greenwashing. They influence consumers to have a positive attitude and give good evaluations regardless of other factors.

Additionally, our findings substantially support our hypothesized model by showing that GBI, and GTR mediate the GW-GBE relationship, and GC moderates between GW and GBE. More specifically, GW is negatively related to GBI, GSA, and GTR which then positively influences GBE. These results are in line with prior studies [1, 33, 77, 83, 123]. Furthermore, the moderating role of GC in the relationship between GW and GBE is confirmed. This means that the higher the GC is, the more adverse effect on GBE the GW will be. In other words, for consumers who have a higher level of GC, the relationship between GW and GBE is weaker, and vice versa. While GC was confirmed as a moderator in the relationship between GW and green purchasing intentions [98], this study is the first to investigate the moderating role of GC in the relationship between GW and GBE in a green marketing context. Simultaneously, our results underline the importance of GBI, GSA, and GTR as both antecedents and major drivers of GBE [1, 2, 25, 33, 59], and the moderating effect of GC in GW-GBE relationship. Therefore, it can be said that GW is a barrier or obstacle to expanding green marketing approaches.

### The mediating roles of GBI and GTR

Our results show that greenwashing adversely affects GBI and GTR and validate the mediating role of GBI and GTR. While a reduction of greenwashing may enhance GBI and GTR, GBI and GTR are, in turn, the key significant mediators connecting greenwashing to green brand equity [see, for example, 1, 2, 25, 33]. Consistent with prior research that has examined the mediating role of GBI, GSA, GTR, green brand association, and brand credibility [see 1, 33, 40], our study contributes favorable evidence with regard to the mechanisms for greenwashing to influence green brand equity. That is to say that firms are encouraged to diminish greenwashing, and thus improve GBI and GTR in order to promote GBE in the context of green marketing.

### The moderating role of GC

Our work is probably the pioneering study for exploring the moderating role of GC in the GW-GBE relationship. GC is negatively associated with GBE. Therefore, GBE is adversely affected by consumers with a high level of environmental concerns. Our empirical findings further verify the moderating effect of GC in the GW-GBE relationship as the influence is different for high versus low levels of GC. This may be because customers with a greater GC are more aware of the actual environmental consequences, and can distinguish substantive greening from a symbolic one. In other words, at a higher level of GC, the relationship is weaker when compared to that of a lower level of GC. Additionally, GC reflects consumers’ opinions of their own environmental stewardship, which motivates people to be aware of their part in resolving environmental issues. This implies that GBE is likely to be reduced by customers with high levels of GC when compared to customers with lower levels of GC.

### Theoretical implications

Our study offers five theoretical contributions. First, in contrast to conventional research, which concentrates on investors, and stakeholders in general, our work which, itself, is grounded in legitimacy and signaling theories, responds to the recent call for research into the consumer repercussion of greenwashing [1] by investigating GW and its role in GBE. Specifically, [1] called for research “on other countries in comparison with this study”. Although our study does not confirm the significant GW-GBE relationship possibly due to the halo effect, it does, however, confirm the full mediating role of GBI and GTR in the relationship between GW and GBE. This provides both a better insight regarding impact mechanisms of GW on GBE via GBI and GTR as well as important evidence toward a unified theory of brand equity [35].

Second, our study sheds light on the understanding of the mechanism in which GW influences GBE. Specifically, our work explores the mediating effects of GBI, GSA, and GTR, as well as the moderating effect of GC in the GW-GBE relationship. GC is often regarded as an antecedent to green brand equity [2, 32], in a mediating role in green practices and green purchase decisions [124, 125], or a moderating role in green purchase intention research [98], yet not in a moderating role in GBE literature. Furthermore, we integrate GBI, GSA, and GTR as mediators into the research framework to study the relationship between GW and GBE in a more systematic manner. Compared with [1], who found the partial mediating role of GBI and GSA, our findings extend our understanding regarding the full mediating role of GBI and GTR in the GW-GBE relationship. Together with [1], our findings validate the different mechanisms, namely, a partial or full mediating role that GBI, GSA, and GTR can play in the GW-GBE relationship in the context of consumers of electronic products.

Third, in line with other research that studies GC in an eco-friendly context, our work is pioneering in that it is the first study to investigate the moderating role of GC in the GW-GBE relationship in a developing country, namely, Vietnam, whose environment is deteriorating as a result a lack of sound regulations during the strong economic growth. Our findings support the ideas that green messages should be provided authentically and sufficiently to potential consumers as they can significantly affect GBE, and eventually, customers’ purchase intentions.

Fourth, our findings both enrich the growing body of green marketing literature and contribute to other fields such as sustainable development as well. Specifically, our work sheds light on how firms should reconsider their greenwashing practices seriously, and then respond sufficiently to consumers’ environmental concerns in order to increase their GBE sustainably, given the high pressure on Vietnam to achieve sustainable development goals in 2030 as has been committed [126]. If consumers realized a discrepancy between green advertising and firm performance, they may earn distrust for, or disqualify the brand image which would result in a decline in GBE. Thus, minimizing greenwashing practices and maximizing GBI and GTR, and strengthening GC may simultaneously and legitimately optimize GBE. This is one way of contributing to sustainable development goals (SDGs), as achieving SDGs strongly needs a systematic and collective effort [127].

Finally, our study’s important contribution is developing and testing the hypothesized model based on the legitimacy and signaling theories. Various contexts have been used to test the suitability of applying signaling theory and legitimacy theory to greenwashing studies [43, 128, 129]. Nevertheless, few studies in the literature on greenwashing combined the legitimacy theory and the signaling theory [43]. The former, which is based on the idea of a "social contract," has typically focused on symbolic actions that could guarantee an organization’s legitimacy [44, 130]. The latter is a theory of economics that discusses how stakeholders look for "signals" that could help them better understand something concerning an unsettled attribute. If information asymmetry exists, for example, the partner with more information may send "signals" to the partner with little information to help them understand or ask them to make judgements [131]. The four factors of GBE that the research model suggests collectively represent the various significant groups of signals. The results demonstrate that, with the exception of greenwash because of the halo effect, GBE is significantly influenced by GBI, GSA, and GTR. Similarly, GW also has a negative impact on GBI, and GTR. On the one hand, these results demonstrate how the signals of interest (GW, GBI, and GTR) affect GBE, which on the one hand add to the signaling theory [50, 51, 53]. On the other hand, the results offer support for the central tenet of legitimacy theory [44]. The more likely it is that consumers will believe a company is greenwashing, the worse it will be for the green brand image, and trust, all of which have a positive effect on GBE. These results confirm those of earlier studies [39, 43] that customers are important authenticators of GW conceptions, and that businesses must genuinely and credibly support their environmental claims. Furthermore, as was already mentioned, this study went beyond [1] by including the GC variable as the moderator in the GW- GBE relationship. Such an addition enhances theory, strengthens comprehension of the role that GC may interact with Vietnamese consumers of electronic goods in an emerging economy like Vietnam, and thereby raises the level of external validity [105, 132–135]. Additionally, employing a cluster random sampling method and attaining a high response rate increases the chances that the results will be externally valid, supporting the theory [105, 107, 136].

### Practical implications

Our work has three important practical implications for businesses, particularly in developing countries to follow green brand equity. First, it is generally agreed that greenwash adversely affects GBI, GSA, and GTR and GBE [1, 40, 81]. Our study demonstrates that the decrease of GW advances GBI and GTR which then boosts GBE. Therefore, in order to strengthen GBE, firms are advised to mitigate or alleviate consumers’ skepticism for their GW and simultaneously augment GBI and GTR. More specifically, firms may need to practice radical transparency in particular to dispel doubts about greenwashing and to disclose information in such a manner that is increasingly thorough, comparable, and reliable. A strong brand equity may assist firms in achieving a better competitive advantage in the marketplace and improve performance. Firms may focus on building brand identity, clarifying which values firms want to bring to the marketplace, or choosing the right media for the right reasons. These are among the many ways for building a stronger brand image for firms. Additionally, firms may want to build better trust by creating relationships that are mutually beneficial, being flexible and patient, and telling the truth to the consumers. These behaviors may help firms to maximize their brand equity. Second, as GBI and GTR are significant mediators in the research model, firms are encouraged to maximize GBI and GTR to enhance their GBE. In other words, firms should broaden and deepen their green practices to their consumers to enhance the extent of the positive relationships between GBI, GTR and GBE. For example, firms may want to improve quality of products and/or price. This may help to improve brand image. At the same time, firms may want to enrich brand trust, for example, by disclosing full information of products on brands, and investing more for word-of-mouth, events or advertising. Improving both brand image and brand trust may inspire or stimulate purchasing intentions or behaviors. Third, our findings indicate that GW is adversely related to GBI and GTR, which is positively related to GBE. This means that GW has an adverse impact on GBE, indirectly via GBI and GTR. These findings help to encourage marketers to make green claims that are more honest and accurate. Finally, when consumers perceive businesses’ greenwashing activities, consumer GC leads to a greater drop in GBE. This means that customers with greater GC levels will be better able to distinguish genuine green claims or items from greenwashing. Therefore, in order to create a fairer market for sustainable development, businesses should promote and educate customers to raise their GC. In the long term, firms should incorporate the visions of GBI and GTR into their long-term planning for green strategies.

## Conclusions

This study examines the impact mechanism of GW on GBE using the legitimacy and signaling theories, focusing on the mediating effects of GBI, GSA, and GTR, as well as the moderating effect of GC. Based on 436 responses from purchasers of electronic products in Vietnam, we determine that GBI and GTR play a full mediating role as greenwashing is not significantly associated with GBE, while GC increases the adverse association between GW and GBE.

### Limitations and implications for future studies

Our research suffers several limitations that need to be acknowledged. First, our work only examines information from—and electronic products in—Ho Chi Minh City, Vietnam. Further research may concentrate on a wider range of other product types in order to have a better understanding of the effects of GW, GBI, GSA, GTR, and GC on GBE. Second, our study adopts the questionnaire-based survey design which captures data at a single point in time to validate the proposed hypotheses. Consequently, it is impossible to identify the dynamic fluctuation of GW, GBI, GSA, GTR, and GC in the various stages of the environmental regulations. Therefore, future research should use a longitudinal design to study the variations of GW, GBI, GSA, GTR, and GC in the various stages of the environmental regulations. Lastly, in the research model, this analysis only considers variables from the perspective of the consumer relationship. It does not investigate consequences of GBE on consumer attitude, or purchase and post purchase behaviors. Future research can incorporate factors from the perceived risk theory and/or investigate from the perspective of consumer loyalty to extend our understanding of effects of GW, GBI, GSA, GTR, and GC in green marketing behaviors.

## Supporting information

S1 File (CSV)
